# Drug Discovery against *Acanthamoeba* Infections: Present Knowledge and Unmet Needs

**DOI:** 10.3390/pathogens9050405

**Published:** 2020-05-22

**Authors:** Hany M. Elsheikha, Ruqaiyyah Siddiqui, Naveed Ahmed Khan

**Affiliations:** 1 School of Veterinary Medicine and Science, University of Nottingham, Sutton Bonington LE12 5RD, UK; hany.elsheikha@nottingham.ac.uk; 2 Department of Biology, Chemistry and Environmental Sciences, College of Arts and Sciences, American University of Sharjah, University City, Sharjah 26666, UAE; rsiddiqui@aus.edu

**Keywords:** *Acanthamoeba*, combination therapy, alternative treatment, drug discovery, natural products, drug repurposing

## Abstract

Although major strides have been made in developing and testing various anti-acanthamoebic drugs, recurrent infections, inadequate treatment outcomes, health complications, and side effects associated with the use of currently available drugs necessitate the development of more effective and safe therapeutic regimens. For any new anti-acanthamoebic drugs to be more effective, they must have either superior potency and safety or at least comparable potency and an improved safety profile compared to the existing drugs. The development of the so-called ‘next-generation’ anti-acanthamoebic agents to address this challenge is an active area of research. Here, we review the current status of anti-acanthamoebic drugs and discuss recent progress in identifying novel pharmacological targets and new approaches, such as drug repurposing, development of small interfering RNA (siRNA)-based therapies and testing natural products and their derivatives. Some of the discussed approaches have the potential to change the therapeutic landscape of *Acanthamoeba* infections.

## 1. Introduction

**Figure 1:**
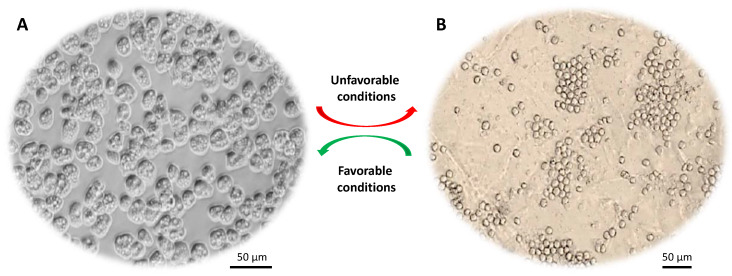
Life cycle of *Acanthamoeba* spp. (**A**) Trophozoite form that divides actively via binary fission. (**B**) Cyst form that represents the dormant stage. Under harsh conditions (e.g., food deprivation, extremes in pH, temperature and osmolarity) trophozoites transform into dormant cysts.

The unicellular eukaryotic *Acanthamoeba* spp. are ubiquitous free-living protists and can survive in various environments, such as water, soil and air. During its life cycle (Figure 1), *Acanthamoeba* can undergo a phenotypic transition, under stressful conditions, from being an active trophozoite to a dormant cyst and vice versa [1]. The trophozoite is irregular in shape with pseudopods for locomotion and spike-like protrusions called acanthopodia [2]. The latter mediate the adhesion of *Acanthamoeba* to biological and inert surfaces [3]. The subcellular features of trophozoite include nucleus, mitochondria, endoplasmic reticulum (ER), Golgi complex, digestive vacuoles, and contractile vacuoles (for osmoregulation), which are enclosed by a thin plasma membrane [4]. The cyst stage, besides being round shaped, is enclosed by a distinctive thick wall, which is made mainly of cellulose [5,6].

*Acanthamoeba* trophozoites (15–45 μm) are metabolically and reproductively active in the presence of appropriate environmental conditions, such as sufficient nutrients and favorable osmolarity, pH and temperature. However, this organism has a remarkable ability to transform into a dormant cyst stage (10–25 μm) under stressful conditions, such as adverse temperature, high osmolarity, high salination, extreme pH and scarcity of nutrients or drought [7]. *Acanthamoeba* cysts have the ability to persist in the environment for many years without losing their virulence and they can be airborne. During this encystation process, *Acanthamoeba* surrounds itself with a unique capsule that consists of two dense layers (inner endocyst and outer ectocyst). A recent study showed that cyst wall proteins are largely composed of three groups of cellulose binding lectins and that cyst wall formation is a well-orchestrated process whereby lectins bind with glycopolymers to form a well-developed cyst wall supported with an endocyst layer [8]. This protective cyst wall is what makes *Acanthamoeba* cysts tolerant to antibiotics and biocides including chlorination [7], and can survive under extreme physiological, radiological and chemical conditions. Once conditions become favorable, cysts switch back to their trophozoite form, a process known as excystation. Cysts are primarily responsible for prolonged treatment of *Acanthamoeba* infections. The majority of available drugs target functional aspects such as synthesis of DNA, RNA, protein, cell wall, or metabolic activity of the pathogen. However, cysts are dormant and exhibit little, if any, of the aforementioned functions, making them resilient to the available drugs. Discovering new anti-acanthamoebic drugs capable of tackling the cystic stage is increasingly difficult partly because the cyst wall is impervious to most drugs and partly because *Acanthamoeba* encysts deep within the corneal stroma [2]. These properties make cysts hard to target effectively. 

Given the opportunity and host susceptibility, pathogenic *Acanthamoeba* is notoriously known to inflict significant damage on the eye and Central Nervous System (CNS) of the affected individuals. Ocular infection by *Acanthamoeba* is associated with a painful condition, known as *Acanthamoeba* keratitis (AK), with potentially sight threatening consequences. The higher cyst density and the presence of a deep corneal ring infiltrate are associated with more severity of AK [9,10]. AK is particularly common in people who wear contact lenses [11]. However, non-contact lens users can be also affected [12]. *Acanthamoeba* can cause a fatal encephalitis, but this form of infection is relatively underrecognized, probably due to its low prevalence and non-specific clinical symptoms.

In this review, we summarize the clinical manifestations of *Acanthamoeba* infection and review the current therapeutic strategies along with the challenges for achieving satisfactory treatment outcomes. In addition, we highlight the key achievements made in the field of anti-acanthamoebic drug discovery, which are expected in the long term to shape the future landscape of treatment of *Acanthamoeba* infection.

## 2. Clinical Features

The opportunistic protozoa *Acanthamoeba castellanii* and *A. polyphaga* can cause severe eye infection, leading to the devastating AK condition. Other *Acanthamoeba* spp., such as *A. rhysodes*, *A. culbertsoni*, *A. hatchetti*, *A. griffini*, *A. mauritaniensis*, *A. lugdunensis* of the T4 genotype, and also genotypes T1, T2, T3, T5, T6, and T11, have been involved in eye infections. Since the early 1970s, this condition has received significant attention from the medical community and the general public in part due to increasing use of contact lenses [13]. Amoebic keratitis can represent a challenge to ophthalmologists because it is often misdiagnosed with bacterial [14] or fungal [15] infection, given the non-specificity of symptoms, particularly during early infection. Any delay in instigating appropriate treatment will cause AK to progress from non-specific symptoms to sight-threatening consequences, with very poor prognosis. Clinical features of AK include blurred vision, eyelid ptosis, agonizing eye pain, photosensitivity, redness of the eye, scleritis, tearing, corneal ulcer, and ring-shaped infiltrates, especially in late stage or in coinfections [2,16]. Complications associated with AK include glaucoma, iris atrophy, anterior synechiae, and cataract. 

*Acanthamoeba* spp., mostly of the T4 genotype, however T1, T10, T12, have been implicated as serious agents of a rare, but invasive brain infection, which is known as granulomatous amebic encephalitis (GAE). This condition often occurs in immunocompromised and debilitated patients with disturbances in the immune system that render them highly susceptible to GAE, such as those with malignancies, AIDS, diabetes, and organ transplant recipients [17,18,19]. Although pathogenesis of GAE is multifaceted and remains largely unknown, the parasite seems to enter the human body via the pulmonary route, and through alveolar blood vessel and hematogenous spread it gains access to the blood–brain barrier (BBB). Early clinical symptoms include headache, nausea, irritability, dizziness and pyrexia, which can overlap with the symptoms associated with viral or bacterial meningitis. As the condition progresses, patients may develop abnormal speech, ataxia, confusion, flaccid paralysis, hallucination, seizures, stiff neck, and unconsciousness [5,18,19,20]. The mortality rate can exceed 90% [21], when the parasites invade and damage the CNS [21] and cause increased intracranial pressure [20]. Severe necrotic brain lesions, including hemorrhaging and edema, can be observed via neuroimaging techniques or on post-mortem examination.

## 3. Current Anti-Acanthamoebic Therapy

**Figure 2:**
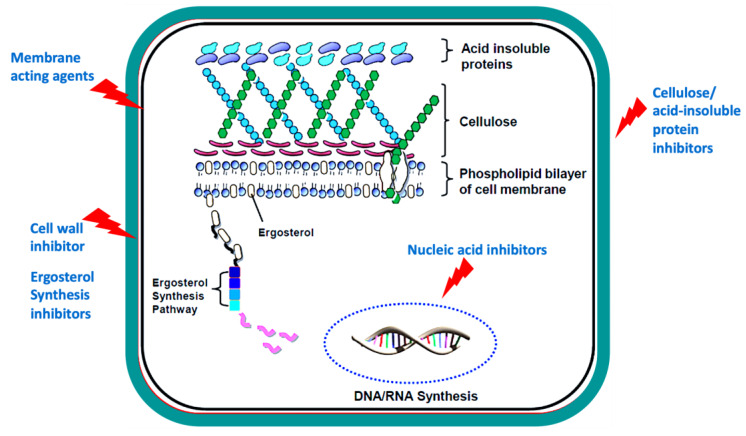
Potential targets used in the development of anti-acanthamoebic agents.

Anti-acanthamoebic drug therapy is the cornerstone of medical management for *Acanthamoeba* infection, which often involves the use of combination strategies to provide synergistic effects and improved treatment outcomes. The treatment regimen normally involves using 0.02% biguanides and 0.1% diamidines [22,23]. Biguanides (Polyhexamethylene (PHMB) or chlorhexidine gluconate (CHX)) represent the first-line therapeutic option and have proven efficacy in curing patients with AK [24]. Biguanides target the plasma membrane of the amoeba leading to membrane damage, lysis, and death. The antiseptic diamidines (propamidine isethionate and hexamidine) are active against the trophozoites and cysts and exert their effects by disrupting the biosynthesis of protein and nucleic acids [25]. An earlier study discouraged the use of propamidine, owing to potential neurotoxicity and resistance of the cysts to the drug [26]. Therefore, combination treatment regimens involving antiseptic agents have been advocated, however, the evidence for the superiority of the combination treatment over monotherapy remains inconclusive [11]. In fact, treatment of AK using 0.02% PHMB had similar efficacy to combined biguanide and diamidine therapy [27]. Effective treatment of AK requires early diagnosis and timely implementation of a strict treatment regimen in order to achieve sufficient eradication of the susceptible trophozoites before they form cysts, which are very difficult to eliminate. The treatment regimen starts with hourly administration of eye drops during the first two days, followed by hourly eye drops per daytime only for the following five days [28]. Given the challenges associated with the management of *Acanthamoeba* infection, significant efforts have been made to develop and test several drugs against *Acanthamoeba*, such as antiseptics, antibiotics and antifungal drugs affecting various targets/functions in *Acanthamoeba* (Figure 2). 

The antiseptics acriflavine and proflavine have activities against trophozoites and cysts, and exert their actions by binding to the nuclear and cellular membranes, and compromising the lipid bilayer and membrane integrity [25]. Phospholipid analogues have been shown to have trophicidal and limited cysticidal activities [29]. They exert their effects by suppressing phospholipid biosynthesis, disruption of cellular membranes and intracellular signalling, and induction of apoptosis [30]. The herbicide 2,6-dichlorobenzonitrile inhibits cellulose synthesis and encystment of the amoeba [31]. The disinfectant povidone-iodine (PVP-I) has both trophicidal and cysticidal effects in vitro [32]. It works by damaging protein and nucleic acid synthesis [33]. In vitro study showed that at 0.5–2.5% PVP-I has a better activity on the trophozoite and cystic stages than CHX [31]. In vitro screening of derivatives of the heterocyclic compounds 1H-benzimidazole and 1H-benzotriazole, and their N-alkylated analogues against *A. castellanii* identified 5,6-dimethyl-1H-benzotriazole and 5,6-dibromo-1H-benzotriazole to be more cysticidal compared to CHX [34]. Also, the antimetabolite, anticancer drugs methotrexate (MTX) and 5-fluorouracil (5-FU) have been shown to inhibit the growth of *A. castellanii* [35].

The antibiotics polymyxin B and polymyxin E (known as colistin) target the plasma membrane of the target organisms, and possess trophicidal and cysticidal activity in vitro [25]. Limitations associated with polymyxins include, nephrotoxicity against human cells, varying efficacies between *Acanthamoeba* spp. and strains, the need to use high concentrations to achieve cysticidal effect [25,36]. The aminoglycoside antibiotics (e.g., paromomycin, neomycin, neosporin) exert their effects by inhibition of protein synthesis [25]. These compounds have efficacy against both cysts and trophozoites in vitro [37,38]. The paromomycin and neomycin are more cysticidal when used together with cryotherapy [37,38]. The macrolide antibiotics (e.g., rokitamycin and spiramycin) perform their actions by inhibiting protein translation [25]. Rokitamycin had trophistatic activity, and trophicidal and cysticidal effects at higher concentrations in vitro [39]. Spiramycin has shown a cysticidal activity, but at a high concentration [38]. The folate synthesis inhibitors (e.g., sulphonamides) are trophistatic and exert their effects via disruption of the synthesis of folate, which is needed for DNA synthesis and cell replication [25]. The anti-infective compound myristamidopropyl dimethylamine (MAPD) has shown cysticidal effect in vitro [40]. 

The polyene antifungal amphotericin B exerts its effect by binding to sterol ergosterol present in the membrane of the fungal cell, and creating pores which result in deploralization and cell leakage [25]. Amphotericin B has shown modest efficacy against trophozoite and cystic stages in vitro [38]. The echinocandin antifungal caspofungin has shown strong activity against the cysts and to some extent, against trophozoites in vitro [41]. Caspofungin exerts its action by inhibiting β1,3-glucan synthase enzyme, leading to inhibition of the synthesis of β1,3-glucan, which represents a key constituent in the inner wall of *Acanthamoeba* cysts [25]. Topical application at low concentrations did not cause any adverse effects in rabbits, but toxicity was observed in human cells at 50 μM concentration [41]. The azole antifungals (e.g., clotrimazole, fluconazole, ketoconazole, miconazole) exert their activities by suppressing ergosterol synthesis in the membrane. Specifically they interfere with sterol synthesis via inhibition of CYP-dependent C-14α demethylase enzyme, which plays a role in converting lanosterol to ergosterol [42]. These azoles possess limited trophicidal and cysticidal activity in vitro [25]. Oral fluconazole has been used for AK treatment, and is probably effective following corneal cryosurgery in invasive keratitis [43]. Systemic ketoconazole seems very effective for treatment of AK [44] and recurrent infections could be controlled by clotrimazole following penetrating keratoplasty [45]. 

To our knowledge, a large number of FDA-approved drugs are available. Examples of such drugs include pyrimethamine, rifampin, 5-flucytosine, co-trimoxazole, sulfadiazine, pentamidine, ketoconazole, fluconazole, itraconazole, azithromycin, amphotericin B, and paromomycin. However, no consensus exists on standard therapeutic management of patients with GAE, and clinical cases reporting successful treatment outcomes remain scarce. The repurposed anticancer and anti-leishmaniasis drug miltefosine can significantly eradicate *A. castellanii* trophozoites at 62.5 µM after 24 h exposure, however higher concentrations 250 and 500 µM were required to achieve the minimal trophocidal concentration for *Acanthamoeba* spp. and *A. lugdunensis*, respectively [46]. A previous study suggested the added value of combining miltefosine with albendazole and fluconazole for treating patients with *Balamuthia* GAE [47]. Additionally, oral voriconazole and miltefosine reduced serological titers and brain lesions in an immunocompetent patient with GAE [48]. These therapeutic advantages make miltefosine a good candidate for inclusion in a combination therapeutic regimen for management of GAE.

## 4. Therapeutic Challenges and Increasing Demands for Better Anti-Acanthamoebic Drugs

Despite the advances in anti-acanthamoebic therapy and the limited available armamentarium of chemotherapeutic agents, management of patients with AK and particularly GAE remains a challenge for health services. Unfortunately, there are many limitations with the current therapeutic medications. Even with combined treatment, outcomes seem to be promising only in the early-presenting cases. Also, most of the topical drugs used for treatment of AK are delivered over extended period of time [49]. The extended treatment duration is inconvenient for patients who continue receiving treatment even after clinical resolution to prevent relapses [2]. Indeed, the requirement of a prolonged treatment course to treat AK has been a central dogma in the management of *Acanthamoeba* infection. In addition, current therapeutics can cause toxic keratopathy [50] and trigger encystation and formation of resistant amoebic cysts [51]. Furthermore, new evidence suggest that microbial coinfections should be suspected in AK cases, which are unresponsive to anti-acanthamoebic therapy [15]. The increasing frequency of coinfections complicate regimens further and require additional therapeutic interventions.

Any delay in diagnosis can cause treatment challenging because it postpones the initiation of timely therapy, which gives sufficient time for the parasite to infiltrate deeper into the corneal tissue, potentially encysting to form fully matured cysts, and becoming less responsive to drug treatment(s) [52] or even form fully developed cysts [11]. Incorrect diagnosis is more likely to occur during the late or advanced phase, where the corneal ulcer of AK often exhibits similar features to keratitis of other etiologies [53], specifically fungal keratitis [54] or herpetic keratitis [55]. Treatment outcomes can be compromised by various factors, such as age, severity of illness, treatment with corticosteroids before the diagnosis of AK, or coinfections. Patients with high AK disease severity at diagnosis that previously used corticosteroids prior to diagnosis are more likely to have a less successful treatment outcome [56]. The correlation between the *in vivo* confocal microscopy-morphological features (IVCM-MF) for determining *Acanthamoeba* cyst density (ACD) and clinical staging of AK on presentation suggests that IVCM-MF can be used to predict the visual outcome in patients with AK [10].

Controversies surround the inclusion of corticosteroids in the treatment plans for AK arise from the lack of a conclusive evidence and from conflicting findings between different studies. On one hand, a link between using steroid eye drops and keratic precipitates during severe *Acanthamoeba* infection has been suggested [57]. Also, steroid treatment has been shown to exacerbate AK in rabbit corneas [58]. On the other hand, treatment regimen for AK that includes corticosteroids has been proposed as a means to reduce the pain and discomfort, limit corneal vascularization [59] and reduce corneal inflammation [60]. Another study showed that adding topical corticosteroid after initiation of anti-acanthamoebic therapy does not seem to worsen the clinical outcomes of AK [61]. Therefore, the beneficial benefits of corticosteroids should be balanced against their potential side effects. Also, patients should continue to receive anti-acanthamoebic therapy after topical corticosteroids are discontinued to avoid relapses [11]. When conservative treatment fails, non-pharmacological surgical interventions, such as amniotic membrane grafts, cryotherapy of the cornea, riboflavin/UVA corneal crosslinking, and keratoplasty can be of value in the management of AK, as a last resort [50]. Unfortunately, despite intensive medical and surgical treatment some advanced AK cases with persistent or recurrent infection may require enucleation of the eye [62].

Biguanide drugs require a prolonged treatment course that can last for several months [63]. Prolonged treatment regimens can induce adverse effects given the cytotoxicity of topical biguanides [64,65]. In fact, ocular health complications associated with progressive AK have been ascribed to cytotoxicity following lengthy topical administration of biguanides and/or chlorhexidine gluconate [11,66,67]. New combination therapeutic regimens are required that can improve treatment efficacy, without exacerbating adverse reactions. One major challenge in finding safer therapeutic substances is the similarity between the eukaryote *Acanthamoeba* and the mammalian host [68]. Discovery of new effective drugs would require identification of compounds that are more selective to *Acanthamoeba*-specific cellular components than to the mammalian cells. Further efforts to identify short-course therapeutic regimens that are highly effective in eradicating *Acanthamoeba* from the affected eye, with the very minimal side effects possible and with the least number of drugs, would be useful not only to improve patient outcome and reduce adverse reactions, but also to reduce the complexity of treatment regimens and enhance the compliance of patients to medication. 

Treatment of patients with GAE is even more challenging than treatment of patients with AK. The mortality rate estimates associated with GAE can be too high [18,19,21] and treatment has been hampered by the scarcity of a reliable drug delivery method and the inability of current drugs to overcome the BBB and penetrate into brain parenchyma in sufficient concentrations to kill the amoeba. Despite the report of many chemical compounds that can block or reduce the growth of the *Acanthamoeba* in vitro, as discussed in the subsequent sections, only a few are likely to realize their full therapeutic potential in the clinical setting.

## 5. Anti-*Acanthamoeba* Drug Discovery

### 5.1. Repurposed Drugs

Recent studies have shown that existing drugs used clinically for other diseases may have the potential to target *Acanthamoeba* infections. With anti-acanthamoebic effects, they can progress to randomized, controlled clinical trials to evaluate their effectiveness against *Acanthamoeba* infections, which would save time and drug development costs. For example, three FDA-approved marketed drugs, amlodipine, loperamide, and prochlorperazine were shown to exhibit potent trophicidal effects [69]. Amlodipine is a dihydropyridine calcium channel blocker used in the treatment of hypertension and angina pectoris. Loperamide is a widely used antidiarrheal drug that acts primarily through activation of opioid receptors. Prochlorperazine, a drug of the same class as trifluoperazine, exhibited potent amoebicidal effects. Haloperidol and prochlorperazine act primarily as dopamine receptor blockers and have been used as anti-psychotic drugs. It was interesting to note that prochlorperazine showed potent trophicidal as well as cysticidal effects, while haloperidol was effective against trophozoites but not cysts. A combination of chlorpromazine and rokitamycin exhibited synergistic trophistatic , trophicidal, and cysticidal activities against *A. castellanii* suggesting their usefulness as chemotherapeutic agents against *Acanthamoeba* infections. The precise mode of action of prochlorperazine against *Acanthamoeba* is unclear but it may involve inhibition of amoeba calcium regulatory proteins, or lipophilic action on the amoeba plasma membrane. Prochlorperazine is thought to exert its anti-psychotic effects by blocking dopamine receptors but also has moderate anti-cholinergic and alpha-adrenergic receptor-blocking activity, as compared to haloperidol, which is a weak anti-cholinergic, muscarinic M1 (silent antagonist) at 10 µM [70]. Another anti-cholinergic agent, procyclidine, which is widely used as anti-parkinsonian agents because of its anti-cholinergic action showed trophicidal effects. Digoxin is a potent inhibitor of the active transport of sodium and potassium across cell membranes and showed trophicidal effects, possibly through a combination of lytic and apoptotic signaling induction. As there is limited availability of effective drugs to treat *Acanthamoeba* infections, clinically available drugs offer potential agents in managing AK and GAE. Repurposing drugs suggests the presence of a pharmacophore with microbicidal activity [71] providing an incentive for further investigation into compounds with similar structures. Exploring novel indications for existing drugs is an attractive short-term strategy offering major savings in development time and expense. Similarly, corifungin (a water-soluble polyene macrolide) and tigecycline (a third-generation tetracycline) were found to reduce *Acanthamoeba* growth (73% and 46% inhibition at 100 µM, respectively) by degenerating cytoplasm architecture and dysfunctioning the mitochondria of *A. castellanii* trophozoites [72,73,74]. Drugs that target G-protein coupled receptors (GPCRs) i.e., dopamine, muscarinic receptors, α- or β-adrenergic receptors and 5HT receptors have also been used due to their critical role in cellular signaling. Inhibition of β adrenergic receptor by propranolol affected *A. castellanii* growth, encystation and viability [75]. Combinations of prochlorperazine plus loperamide, prochlorperazine plus apomorphine and procyclidine plus loperamide were proved to be amoebicidal against *A. castellanii* [21]. Moreover, chloroquine, an anti-malarial drug, was able to inhibit autophagy, a type of programmed cell death (PCD), which was highly stimulated during encystation of *Acanthamoeba*, leading to reduction in the survival of *A. castellanii* [73]. Atorvastatin, fluvastatin, simvastatin and voriconazole could also induce PCD in *A. castellanii* [76].

### 5.2. Improvements in Existing Drugs

The reformulation or analogues of drugs can improve compliance, pharmacodynamics and pharmacokinetics, making current medicines more clinically effective. High throughput screening of structural analogues of miltefosine such as heterocyclic alkylphosphocholines (APCs) [77] and oleylphosphocholine (OlPC) [78] demonstrated similar or stronger *in vivo* efficacy compared to miltefosine. It is worth noting that heterocyclic alkylphosphocholines possess ability to cross the BBB [79] and have potential in treating GAE due to *Acanthamoeba* and possibly other brain-infecting amoebae. Recently several studies have shown that the efficacy of marketed drugs can be enhanced by conjugation with metals such as gold or silver to synthesize drug-conjugated metal nanoparticles and can be repurposed as potential drugs for treating infections due to pathogenic free-living amoebae [80]. However, the use of metal nanoparticles can be associated with cytotoxicity [81] and side effects [82]. 

To develop a holistic approach to control AK certain measures should be included to augment pharmacotherapy, for example, by reducing incidence of eye infection by including new compounds with proven efficacies against trophozoites and cysts in cleaning solutions of contact lenses. In this regard, a recent study [83] showed that quaternary ammonium compounds (QACs) are more potent than APCs and had cysticidal activity against matured cysts at IC*_50s_* 19.00  ±  0.03  µg/mL and 15.00  ± 0.06  µg/mL for QAC16, and QAC18, respectively. QAC with 12 alkyl carbon chain (QAC12) increased the biomass of trophozoites, delayed encystation by 96 h, but failed to trigger excystation. However, QAC12 potentiated the toxicity of APC16 against trophozoites. The toxicity of QACs was related to the length of the alkyl-carbon chain and was achieved by producing permeabilization and DNA complexing in trophozoites. The effects of combining atorvastatin (a statin used to lower blood cholesterol) with two eye drops (Optiben and Diclofenaco-lepori (D-L)) against *A. castellanii* and on the viability of a murine macrophage were investigated. The ideal combination that reduced the parasite growth without causing cytotoxicity was 30% Optiben, 63.5% atorvastatin, and 3.1% water. In addition, the most effective combination that inhibited the parasite growth with limited cytotoxicity was 17.6% Diclofenaco-lepori and 82.4% atorvastatin [84]. Another study reported low EC_50_ values for prodigiosin (2.2 μM) and obatoclax (0.5 μM) against *A. castellanii* trophozoites [85].

### 5.3. siRNA-Based Therapeutics

Small interfering RNA molecules (siRNAs) have highlighted the benefits of existing statins against AK [86]. siRNA molecules can be synthesized artificially to silence or knockdown a particular mRNA. They could be used as a potent therapeutic option or a method for target validation in drug discovery. Specific targets investigated include extracellular serine protease [87], xylose isomerase [88], encystation-mediating serine proteinase (EMSP) [89], protein kinase C [90], cellulose synthase [91], protein arginine methyltransferase 1 [92], cysteine protease inhibitor (AcStefin) [93], and 3-Hydroxy-3-methylglutaryl-coenzyme A (HMG-CoA) [86]. It was found that after silencing the aforementioned targets by siRNA, encystation was prevented. It was also proposed that a combination of two gene-specific siRNAs (one targeting serine proteases and one targeting glycogen phosphorylase) could affect the growth rate and survival of amoeba [94]. Later, Zorzi et al. [95] designed siRNA-loaded liposomes for the successful treatment of a murine model of ocular keratitis caused by *Acanthamoeba*, further highlighting siRNAs as a promising future therapeutic approach. 

### 5.4. Alternative Therapeutics

The effectiveness of a murine monoclonal anti-idiotypic antibody and a synthetic killer mimotope (resembling a yeast toxin) in inhibiting and damaging *Acanthamoeba* growth on contact lenses has been shown [96]. Therefore, these biologics show clinical potential for development in order to prevent *Acanthamoeba* growth on contact lenses. These would consist of an *A. castellanii*-specific Fab portion that is specific for its surface, covalently linked to the A chain of the diphtheria toxin [97]. Similarly, photodynamic chemotherapy is a novel intervention that involves the use of *Acanthamoeba*-specific antibodies linked to photosensitizers like phthalocyanine (RLP068) or Hypocrellin B [53]. Photodynamic therapy may be advantageous over conventional methods. It is a treatment that involves the use of light-sensitive medication and a light source to destroy cells. Incubation of cysts with compounds and irradiation with 600–700nm light has been found to cause rapid and extensive damage, but its clinical validity is yet to be confirmed [98]. Photochemotherapeutic strategy was proposed to target *Acanthamoeba* infections. As mannose-binding protein is expressed on the surface membranes, photosensitizing compound porphyrin conjugated with mannose could achieve more specific drug targeting. Pre-treatment with this could reduce host cell cytotoxicity from 97% to 4.9% [99]. A recent study showed that Rose bengal photodynamic antimicrobial therapy can reduce parasitic load and diminished clinical severity of AK in a rabbit model [100].

### 5.5. Natural Compounds

Traditionally many drugs with biological activity were discovered from natural compounds. Among a plethora of natural compounds tested, a few examples include *Ipomoea* spp., *Kaempferia galanga*, *Cananga odorata* [101], oakmoss (a natural fragrance ingredient) [102], a hexane fraction of *Pterocaulon polystachyum* (Asteraceae) [103], ethyl acetate extract of Limouni olive leaf [104], resveratrol and curcuminoids [105] were found to be amoebicidal. Of note, resveratrol is of high interest for further investigation because it can prevent amoeba binding to the human brain microvascular endothelial cells (hBMECs) and it is selective to *A. castellanii*, but not the hBMECs [105]. Interestingly, resveratrol could also act as a topoisomerase II inhibitor [106] that prevents DNA ligation and subsequently leads to cell apoptosis. Plant extracts from *Rubus chamaemorus*, *Pueraria lobate* and *Solidago virgaurea* could also be used in combination with other GAE drugs as they were found to extend the survival of *Acanthamoeba*-infected animals [107]. 

Plant-derived artemisinin and artesunate have caused 93% reduction in the trophozoite growth [108]. They exert their amoebistatic activity via induction of reactive oxygen species and lipid peroxidation, leading to oxidative stress and apoptosis [25]. Magainins, peptides produced by the skin of the African frog, had both trophistatic and trophicidal activities in vitro. Their anti-acanthamoebic effects are attributed to interruption of the ion conductance across the cell membrane [109]. A recent study showed that a number of natural compounds of plant or commercial origins (e.g., quercetin, kolavenic acid extracted from plant *Polyalthia longifolia* var pendula and crude plant methanolic extract of *Caesalpinia pulcherrima*) exhibited considerable suppression of the amoebae growth [110]. Conjugation of the plant-derived compounds (e.g., quercetin) with silver nanoparticles increased their anti-acanthamoebic effect, and reduced the encystation and excystation of *A. castellanii*, without exhibiting toxicity against human cells [110]. Taken collectively, this broad range of nutraceuticals display promising anti-acanthamoebic potential, making natural products interesting drug leads in the foreseeable future. 

### 5.6. Potential Targets for New Anti-Acanthamoeba Therapy

Many pharmacological targets have been discovered over the last few years [30]. For example, the myosin superfamily includes 18 different classes of motor proteins. Of the many classes that are expressed in *Acanthamoeba*, myosin-I and -II have been studied most extensively. The function of both myosins is based on the use of ATP hydrolysis to generate forces required for cellular functions. *Acanthamoeba* expresses three types of myosin-I subtypes, myosin-IA, -IB, and -IC. Myosin-IA functions in cytoplasmic vesicle transport, myosin-IB functions in pseudopod extension and phagocytosis, and myosin-IC is the only subtype that functions in contractile vacuole [25]. *Acanthamoeba* myosin-IC was of particular interest as it performs functions that human myosin-IC lacks and it is only 44% homologous to human myosin-IC [111]. The contractile vacuole is highly important in *Acanthamoeba* because it maintains homeostasis by regulating the amount of water within amoeba. It absorbs water by osmosis from the cytoplasm and moves to the surface of the amoeba and undergoes exocytosis when full. If myosin-IC is blocked, *Acanthamoeba* would be unable to regulate its internal water content and ultimately lead to cell lysis. Pentachloropseudilin (PCIP), a non-competitive, reversible myosin-IC inhibitor, is the only specific myosin-IC inhibitor developed and tested on Hela cells [112] but has not been tested on *Acanthamoeba*. It works by reducing the coupling between actin and nucleotide blinding sites [111]. Looking at the effects of PCIP analogues to inhibit or kill trophozoites might provide additional insight to current therapy. 

Moxifloxacin has shown limited efficacy against *Acanthamoeba* and exerts its effect by inhibition of DNA gyrase, a type II topoisomerase, and topoisomerase IV, which is required for DNA replication. Topoisomerase I inhibitors (e.g., camptothecin, irinotecan, topotecan) and topoisomerase II inhibitors (e.g., doxorubicin, amsacrine, etoposide) are generally used as anti-cancer drugs to induce apoptosis and death of cancer cells, and may have a potential activity against *Acanthamoeba*. High levels of elastase activity were found in *A. culbertsoni* [18]. Hence, it will be important to examine the potential of elastase inhibitors to inhibit *Acanthamoeba* elaborate elastase, a type of protease that degrades connective tissue proteins and causes cellular damage. The majority of the cyst wall structure is cellulose; thus, targeting cellulose biosynthesis can prevent encystment. The 2,6-dichlorobenzonitrile (DCB), a cellulose synthesis inhibitor, blocked *Acanthamoeba* encystment [113]. Using cellulase to the degrade cyst wall might make amoeba more susceptible to therapeutic compounds [114]. Alkaline phosphates have been identified in *Acanthamoeba* contractile vacuoles [115] and can be targeted by compounds, such as polyoxometalates. *A. castellanii* was found to have a novel complement of shikimate pathway enzymes [116]. Using (6S)-6-fluoroshikimic acid (antibacterial) and glyphosate (herbicide) can be a new approach to inhibit the shikimate pathway enzymes.

Agents that affect membrane sterols, which are present in trophozoites and cysts, but absent from the host cells, have the potential to selectively suppress the amoeba growth. This assumption motivated Shing et al. [117] to examine the anti-acanthamoebic potential of the FDA-approved antifungal conazoles, which target sterol 14-demethylase (CYP51). Isavuconazole and posaconazole showed high efficacies against *A. castellanii* trophozoites. Additionally, isavuconazole damaged trophozoites within a day and suppressed excystation. Given the high safety of isavuconazole and its ability to block *A. castellanii* excystation, this drug was suggested as a cost-effective option for the treatment of primary and repeated AK. Reyes-Batlle et al., [118] identified new N-substituted quinolin-2(1H)-ones compounds with selective toxicity against trophozoites and cysts. The compounds’ toxicity was attributed to their ability to significantly lower the levels of ATP, without increasing the permeability of the cell membrane, leading to apoptosis and death of the amoeba. The promising potential of N-acyl substituted quinolin-2(1H)-ones suggests that these compounds may serve as a new scaffold for the identification of novel and better anti-acanthamoebic drugs. 

### 5.7. New Anti-Acanthamoebic Approaches 

The application of riboflavin (B2) and concurrent ultraviolet light A (UVA) exposure to the cornea has shown potential as a new approach for AK therapy. UV irradiation of B2 produces free radicals that cause oxidation and cross-linking of the corneal collagen [119,120]. This prevents further tissue damage and parasitic reproduction by damaging their nucleic acid material [121]. Despite the potential, there has been no confirmation from clinical trials to incorporate this as a mainstay therapy. The use of drug-carriers improves the penetration of existing drugs into cystic forms in ocular or nasal drug administration. Current drugs; propamidine isethionate 0.1%, neomycin 1% or miconazole 1% lack cysticidal activity, however when combined with dimethyl-sulfoxide 30%, propamidine isethionate 0.1% exhibited better cysticidal activity. As dimethyl-sulfoxide itself has been used topically in the past, it can be considered clinically safe [122]. Furthermore, liposomal carriers of pentamidine isethionate improve the drug’s potency in vitro [123] and chitosan microspheres have been found to improve rokitamycin’s anti-amoebic activity and dissolution rate, providing a controlled-drug release [124]. More recently, drug conjugation with metals such as gold or silver to synthesize drug-conjugated metal nanoparticles have shown tremendous potential in the improved killing of parasites in vitro [80]. 

### 5.8. Improved Drug Delivery to the Blood–Brain Barrier

Effective treatment depends heavily on the drug’s ability to cross the BBB as drug transport is often hampered by highly selective BBB. The majority of drugs that target the brain in clinical practice are lipid soluble small molecules (i.e., antibiotics) with molecular weight < 400 Da [125]. Among all the drugs that were used to treat GAE, none were delivered specifically to the CNS and limited research is done to improve their delivery to the BBB. Although a liposomal delivery is an available drug delivery option for amphotericin B (Ambisome) to enhance its lipid solubility, it was generally unable to cross the BBB [126]. In addition, despite the presence of rifampin and pyrimethamine in effective concentrations in the CSF (another gateway of drug transport to the brain), they transport poorly to the brain [125]. The development of improved drug delivery methods for current anti-acanthamoebic drugs is crucial to enhance the action of existing drugs until novel compounds with increased potency are available.

Four main approaches are currently used to deliver drugs to the CNS [25]: i) transcranial drug delivery (injection in the cranium), ii) intranasal drug delivery that provide direct drug transport to the CSF, iii) transient modification of BBB i.e., increase BBB permeability using ultrasound or electromagnetic heating of nanoparticles [127] and iv) modification of physiochemical properties of drugs such as the lipidization of small molecules, lipid-based nanotherapeutics for the delivery of siRNA, and drug-conjugation with metals such as gold or silver to form drug-conjugated metal nanoparticles [128] and microspheres encapsulation to improve and prolong in vitro anti-amoebic activity. An example of microsphere was rokitamycin, an anti-acanthamoebic macrolide, loaded in microspheres showed better solubility, penetration, and enhancement [124].

### 5.9. Theranostics as A Potential Strategy 

The theranostic approaches, which combine therapeutic and diagnostic methods in one platform, have the potential to overcome conventional diagnostic and therapeutic limitations associated with the management of neglected diseases such as *Acanthamoeba* infections. Recent studies have proposed the theranostic strategy against infections due to pathogenic free-living amoebae [129]. It is hoped that such development can help expedite timely and sensitive diagnosis augmented with effective therapeutic capabilities. However, it requires development of smart materials for improved laboratory and point-of-care testing. Nanomaterials have already shown promising theranostic applications in non-communicable diseases and these can provide a breakthrough against *Acanthamoeba* infections. 

## 6. Conclusions

In recent years, considerable advances have been made in identifying new molecular targets with novel mechanisms of action for the treatment of largely neglected *Acanthamoeba* infections. Although concerns remain regarding the lack of drugs with high anti-acanthamoebic efficacy and low toxicity, the potential of some existing medicines to be repurposed for anti-acanthamoebic indication is being explored to address these issues. In particular, the theranostic approach is considered as a highly valuable approach in targeting infections caused by pathogenic amoebae. Significant efforts are still needed to employ alternative or adjunct treatment approaches for the development and evaluation of more effective and safer therapeutic modalities. In this review, we discussed key research areas that can bolster the anti-acanthamoebic drug pipeline and can, in the long term, tackle the current unmet clinical needs in the treatment of *Acanthamoeba* infection.
